# Oxidative Stress Induces Caveolin 1 Degradation and Impairs Caveolae Functions in Skeletal Muscle Cells

**DOI:** 10.1371/journal.pone.0122654

**Published:** 2015-03-23

**Authors:** Alexis Mougeolle, Sylvie Poussard, Marion Decossas, Christophe Lamaze, Olivier Lambert, Elise Dargelos

**Affiliations:** 1 Univ Bordeaux, Chimie et Biologie des Membranes et Nanoobjets, UMR 5248, F-33600 Pessac, France; CNRS, Chimie et Biologie des Membranes et Nanoobjets, UMR 5248, F-33600 Pessac, France; Bordeaux INP, Chimie et Biologie des Membranes et Nanoobjets, UMR 5248, F-33600 Pessac, France; 2 Institut Curie—Centre de Recherche, Membrane Dynamics and Mechanics of Intracellular Signaling Team, INSERM U1143, CNRS UMR 3666, Paris, France; Institut de Myologie, FRANCE

## Abstract

Increased level of oxidative stress, a major actor of cellular aging, impairs the regenerative capacity of skeletal muscle and leads to the reduction in the number and size of muscle fibers causing sarcopenia. Caveolin 1 is the major component of caveolae, small membrane invaginations involved in signaling and endocytic trafficking. Their role has recently expanded to mechanosensing and to the regulation of oxidative stress-induced pathways. Here, we increased the amount of reactive oxidative species in myoblasts by addition of hydrogen peroxide (H_2_O_2_) at non-toxic concentrations. The expression level of caveolin 1 was significantly decreased as early as 10 min after 500 μM H_2_O_2_ treatment. This reduction was not observed in the presence of a proteasome inhibitor, suggesting that caveolin 1 was rapidly degraded by the proteasome. In spite of caveolin 1 decrease, caveolae were still able to assemble at the plasma membrane. Their functions however were significantly perturbed by oxidative stress. Endocytosis of a ceramide analog monitored by flow cytometry was significantly diminished after H_2_O_2_ treatment, indicating that oxidative stress impaired its selective internalization via caveolae. The contribution of caveolae to the plasma membrane reservoir has been monitored after osmotic cell swelling. H_2_O_2_ treatment increased membrane fragility revealing that treated cells were more sensitive to an acute mechanical stress. Altogether, our results indicate that H_2_O_2_ decreased caveolin 1 expression and impaired caveolae functions. These data give new insights on age-related deficiencies in skeletal muscle.

## Introduction

Aging is characterized by the deterioration of many physiological functions leading to the development of multiple diseases (cardiovascular and neurodegenerative diseases, diabetes, cancer…). Aging of the skeletal muscle (i.e. sarcopenia) comes with an involuntary and physiological loss of muscle mass and strength [1, 2]. It affects all elderly after the age of about 50, regardless of their overall health condition. Sarcopenia can deprive people of their functional independence, and increase their risk of falls and fractures [3]. With the constant extension of lifespan in the western civilizations, sarcopenia will dramatically impact on quality of life and place ever-increasing demands on public health care [4].

Sarcopenia is a multifactorial syndrome probably resulting from a nutritional and hormonal imbalance and a lack of physical exercise occurring with age [5, 6, 7]. At the cellular level, the reduction in the number and size of muscle fibers could be explained by the impairment of muscle regeneration, i.e. alteration of myogenic regenerative cells or satellite cells and deregulation of the differentiation process [8].

Oxidative damage has been proposed as one of the major contributors to the skeletal muscle aging, this organ being the most oxygenized of the body [9]. Recently, increased reactive oxygen species (ROS) accumulation has been clearly shown in skeletal muscle of old mice [10]. Among the pleiotropic effects of ROS intracellular accumulation, a failure of myogenic regenerative process has been clearly indicated [11].

Caveolae are 50–100 nm invaginations of the plasma membrane with a lipid composition rich in cholesterol and sphingolipids strongly related to lipid rafts [12]. Caveolin (21 to 23 kDa), the main constituent of caveolae has three isoforms. The caveolin 1 and 2 are co-expressed in many tissues and in particular in differentiated cells such as endothelial cells, adipocytes, fibroblasts and type I pneumocytes, while caveolin 3 is a muscle-specific protein. Caveolins 1 and 3 as well as a specific lipid environment (cholesterol, glycosphingolipids) are required for the formation of caveolae. More recently, another family of cytoplasmic proteins has been identified as key regulators of caveolae formation. Cavins would stabilize caveolin oligomers at the plasma membrane [13]. Recently, it was shown that caveolin assembles with cavins to organize a distinct coat around the caveolar bulb [14].

Caveolae have been linked to multiple functions including vesicular transport, cholesterol and calcium homeostasis…More recently caveolae have been shown to constitute a plasma membrane “reservoir” that is mobilized under mechanical stress conditions [15]. Through their Caveolin Scafolding Domain (CSD), caveolins would allow specific interactions with signaling effectors localized in caveolae and would activate or inhibit their signaling activity. This would allow caveolae to act as “cell signaling platforms” [16].

In skeletal muscle, caveolae play a specific role related to the differentiation of myogenic regenerative cells and maintain the contractile unit of differentiated muscle. High numbers of caveolae have been identified in muscle fibers from Duchenne muscular dystrophy, whereas mutations in the caveolin 3 gene cause multiple forms of muscle pathologies [17, 18]. Although caveolae have been largely linked to muscle physiology, little is known about the potential role of the caveolins / caveolae in the etiology of sarcopenia. However, caveolae internalization has been recently shown to trigger plasma membrane repair in muscle fibers [19] and caveolin 1 interaction with Nrf2 transcription factor would be involved in the regulation of cell antioxidant defenses [20]. Caveolae / caveolins could therefore be implicated in the regulation of cellular processes associated with skeletal muscle aging.

In this paper, we showed that oxidative stress induction by addition of hydrogen peroxide (H_2_O_2_) results in Caveolin 1 rapid degradation in proliferative mouse myoblasts. Surprisingly, the decrease in caveolin 1 did not result in decreased caveolae assembly at the plasma membrane. However, the induction of oxidative stress severely impaired two classical caveolae-specific functions: endocytosis and adaptation to mechanical stress. Altogether, these data indicate that Caveolin 1 and caveolae-dependent functions are involved in the mediation of oxidative stress-induced signaling pathways in muscle cells.

## Materials and Methods

### Materials

DMEM, fetal bovine serum, penicillin/streptomycin, trypsin / EDTA, PrestoBlue Cell Viability Assay and Bodipy-Lactosylceramide-BSA were purchased from Life Technology. DCFDA cellular reactive oxygen species detection assay kit was purchased from Abcam. The Protein Carbonyl Assay kit was from Cayman Chemical Company. ECL Prime Western Blot detection kit was from GE Healthcare. Rabbit anti-caveolin 1 and anti-cavin 1 were purchased from Abcam. Anti-p38 α and anti-calnexin antibodies were from Santacruz Biotechnology. Anti-golgin antibodies were from Molecular probes and mouse anti-Ubiquitin antibodies from Calbiochem. Endocytosis specific inhibitors were purchased from Merck. The caveolin 1 specific siRNA, the negative control, lipofectamine and Opti-MEM were purchased from Invitrogen. Alexa-fluor conjugated secondary antibodies were from Invitrogen also. All other chemicals were from Sigma.

### Cell culture

The C_2_C_12_ mouse myoblast cells were obtained from the American Type Culture Collection (Rockville, MD, USA). They were seeded at a density of 6000 cells per cm^2^ in Dulbecco modified Eagle's minimal essential medium (DMEM) supplemented with fetal calf serum (10%) and penicillin / streptomycin (50 U/mL, 50 μg/mL) at 37°C in a humidified incubator (5% CO_2_). Cells were allowed to reach 80% confluence before being treated as described below.

### Western blotting

Cells were collected in PBS Buffer (137 mM NaCl, 2.68 mM KCl, 4 mM Na_2_HPO_4_, 1.76 mM KH_2_PO_4_ pH 7.4 and a protease inhibitor tablet cocktail) and disrupted by brief sonication. Protein content was quantified with Bradford assay.

The samples (15 μg of protein) were applied onto 10% SDS-PAGE according to the Laemmli procedure [21]. Fractionated proteins were then transferred onto an Immobilon membrane. The membranes were blocked for 2 h at room temperature with 5% (w/v) skimmed milk in Tris-Buffered Saline / 0.1% Tween 20 (TBS: 50 mM Tris buffer, pH 8, 138 mM NaCl, and 2.7 mM KCl). After washing in TBS-T, the membranes were incubated for 2 h at room temperature in TBS-T containing the primary antibody at appropriate concentrations and 1% skimmed milk. Antibody dilutions were used as follows: 1:250000 for caveolin 1, 1:1000 for p38 α, 1:1000 for cavin 1, 1:1000 for golgin, 1:250 for calnexin and 1:300 for ubiquitin. The membranes were next washed three times in TBS-T and the peroxidase conjugated anti-rabbit or anti-mouse secondary antibodies were applied (1:50000). Antibody binding was visualized by the addition of ECL Prime detection kit as described by the manufacturer. For relative quantification, the level of p38α was used as a loading control as usually done in other studies related to oxidative stress [22, 23, 24].

### Cell survival analysis

Myoblasts were seeded in 96 well plates (25000 cells / well). The following day, cells were incubated with H_2_O_2_ (500 or 1000 μM) during 10 min to 6 h. When necessary, cells were incubated 3h with 50 μM MG132 before 500 μM H_2_O_2_ was added to the culture medium and left during 10 min. A DMSO control was included since MG132 was prepared in this solvent. To determine cell survival, PrestoBlue Cell Viability Assay was carried out following the manufacturer’s protocol. Cells were rinsed with PBS and incubated 1 h at 37°C in the reagent diluted in DMEM (1/10). Fluorescence intensity was measured with a fluorescence plate reader (TRIAD LT detector from Dynex; λ_exc_ = 560 nm; λ_em_ = 590 nm). Each condition was done in triplicate.

### Intracellular ROS accumulation

Intracellular reactive oxygen species were measured with the cell-permeant fluorogenic dye 2’,7’-dichlorofluorescein diacetate (DCFDA) as recommended by the manufacturer. Myoblasts were plated at a density of 25000 cells per well in 96 well plates in DMEM without phenol red. The following day cells were labeled with DCFDA (25 μM) 45 min at 37°C and treated with 0, 500 or 1000 μM H_2_O_2_ for 10 min to 1 h. Fluorescence intensity was measured with a fluorescence plate reader (TRIAD LT detector from Dynex; λ_exc_ = 485 nm; λ_em_ = 535 nm). Each condition was done in triplicate.

### Carbonyl protein assay

Protein carbonyls were determined by measuring the reactivity of carbonyl derivatives with 2, 4-dinitrophenylhydrazine (DNPH) with the Protein Carbonyl Assay kit as described by the manufacturer. After 3 h treatment with H_2_O_2_, cells were scraped out, briefly sonicated and centrifuged 15 min at 10000g. Supernatants were incubated with DNPH for 1h in the dark. Proteins were precipitated with TCA, washed with ethanol-ethyl acetate (1: 1) and suspended in guanidine hydrochloride. After a 10000g centrifugation, supernatants were disposed in 96 well plates and absorbance was measured at 370 nm. Each condition was done in duplicate.

### Sucrose density fractionation

Caveolae were isolated as described previously [25] with minor modifications. Six 100 mm culture dishes were used for each sucrose gradient. Cells were washed twice with ice-cold PBS and scraped. They were collected by centrifugation (1000*g*), dispersed in 300 μl cold buffer A (10 mM HEPES, pH 7.4, 1 mM MgCl_2_, 1 mM DTT, 5 mM NaN_3_ and protease inhibitor cocktail) supplemented with 250 mM sucrose and containing 1% Triton X-100. Cells were disrupted with a 22 G needle (20 strokes). The homogenate was then adjusted to 45% sucrose by addition of 2.16 M sucrose prepared in buffer A. The sample (890 μL) was transferred to the bottom of a 5 ml centrifuge tube that contained two layers of 5 and 30% sucrose (625 μL and 3,48 mL respectively). After centrifugation (17 h, 200000*g*, in a Beckman MLS-50 rotor at 4°C), ten fractions of 0.5 mL were collected from the top of the gradient to the bottom and used for further analyses.

Fraction 2 of the sucrose gradient was diluted tree times in buffer A and centrifuged at 100000g for one hour. The caveolae fraction was isolated from the resulting pellet and used for immune electron microscopy as indicated below.

### Electron microscopy

Immune electron microscopy (IEM) was performed on vesicles deposited on carbon coated nickel grids submitted to a glow discharge (Elmo, Cordouan). Briefly, grids were labelled with anti-caveolin 1 antibodies (1:50) followed by the protein-A coupled to 10 nm colloidal gold particles (CMC Utrecht). They were finally negative stained using 4% uranyl acetate.

For myoblast observations, cells were embedded by conventional method in resin. Briefly, they were fixed using 1.6% (v/v) glutaraldehyde, post-fixed using 1% (v/v) osmium tetroxide, dehydrated in ascending series of ethanol dilutions and in propylene oxide and embedded in Epon (Inland Europe). Ultra-thin sections (70 nm) (RMC, powertome PC) were collected on butvar-coated single-slot copper grids and stained with 2% (v/v) uranyl acetate and with lead citrate. IEM was performed for caveolin 1 detection using the preembedding immunogold technique according to Decossas et al. [26]. Cells were fixed using 4% formaldehyde during 1 h, incubated in glycine 50 mM and permeabilized using saponin 0.1% for 3 min. Cells were blocked using 1% BSA, incubated in a polyclonal anti-caveolin 1 antibody 20 μg/ml, and then in goat anti-rabbit IgGs conjugated to ultra-small gold particles (0.8 nm in diameter; Aurion; 1:100 in PBS + 2% acetylated BSA). Cells were then post-fixed in 1% glutaraldehyde and finally the diameter of the gold immunoparticles was increased by using a silver enhancement kit (HQ silver; Nanoprobes). After post-fixation in 1% osmium tetroxide for 10 min, cells were embedded in Epon as described previously. Observations were done with a CM120 TEM microscope (FEI) using 2k x 2k USC1000 slow-scan CCD camera (Gatan).

### Epifluorescence and TIRF microscopy

C_2_C_12_ cells grown on glass coverslips were washed three times with PBS and fixed for 15 min with paraformaldehyde (4% w/v in PBS). Cells were then permeabilized for 5 min with PBS containing 1% Triton X-100 and blocked in PBS containing 3% BSA for 1 h. Caveolin 1 antibodies (dilution 1:300) were incubated during 3h in PBS with 1% BSA. Cells were rinsed three times before addition of Alexa-conjugated anti-rabbit antibodies (dilution 1:1000). After being washed, cells were incubated for 5 min with Hoechst (diluted 1:1000 in PBS) washed and placed in mounting medium (Dako). Cells were observed on epifluorescence microscope using Leica DMI6000B with CCD camera (6.45 μm pixel dimension, 1392 x 1040 pixels, Leica) or using a Nikon Ti-Eclipse with EM CCD camera (16 μm pixel dimension, 512 x 512 pixels, Photometrics Evolve) for TIRF images.

### Caveolae-mediated endocytosis

Cells were seeded at a density of 15 000 cells /cm^2^ in 12 wells plates and allowed to adhere for 24 h in growth medium before analysis. One hour after H_2_O_2_ treatment, cells were rinsed once with DMEM without phenol red, incubated with 0.25 μM Bodipy-Lactosylceramide-BSA during 30 min at 10°C, rinsed twice with DMEM on ice and incubated 10 min at 37°C to induce endocytosis. To measure the energy-independent uptake, cells were pre-incubated for 30 min in DMEM without glucose supplemented with 10 mM sodium azide and 20 mM 2-deoxy D-glucose prior to Bodipy LacCer exposure. To selectively inhibit endocytosis routes, cells were pre-incubated 30 min with 15 μM chlorpromazine (clathrin-mediated endocytosis), 200 μM genistein (caveolae-dependent endocytosis) or 5 μM cytochalasin D (actin-dependent macropinocytosis) before Bodipy LacCer addition. Fluorescent lipids potentially present on the cell surface were removed by three washes of 10 min with delipidated BSA at 10°C. Cells were immediately trypsinized and collected for flow cytometry analysis.

Samples were analyzed on a BD Biosciences CANTO flow cytometer with a DIVA software and Bodipy LacCer was excited by a 488 nm laser. Cell debris were excluded, based on the forward and side scatter characteristics of the cell population. The results are expressed as the percentage of the mean of cell fluorescence intensity by analyzing 10 000 cells in the gate. Experiments were carried out on cell samples from three independent cultures.

### Osmotic swelling

When cells reached 70% confluence, H_2_O_2_ (500 μM) or methyl-beta-cyclodextrin (5 mM) were incubated during 10 or 30 min respectively in growth medium. After being rinsed three times with PBS, the cells were left in hypo-osmotic medium. In order to obtain 30 mOsm, sterile water was added to the culture medium (9/1 (v/v)). After 5 min incubation, cells were observed under a light microscope to assess the swelling of the cells. After 30 min, cells were detached from the plates by trypsin treatment and collected by a brief centrifugation. Cell suspensions were mixed with an equal volume of 0.4% trypan blue. The number of unstained/stained cells was counted with a Hematocytometer. Experiments were done in triplicate.

### RNAi

The siRNA oligomers targeting caveolin 1 (Stealth RNAi Cav1MSS273502) were purchased from Invitrogen and transfected into C_2_C_12_ mouse myoblasts at 25 nM using Lipofectamine RNAiMAX in Opti-MEM medium (Invitrogen) according to the manufacturer’s recommendations. Non-targeting siRNA (Stealth RNAi negative control duplexes, Invitrogen) was used as control siRNA. Experiments were carried out 72 h after transfection.

### Statistical analysis

All data are expressed as means ± SEM and are representative of an average of at least three separate experiments. Each single experiment was conducted on separate cultures. The statistical significance of the difference between groups was determined using an unpaired Student’s t test and a Mann-Withney test using XLSTAT software. A value of *P* < 0.05 was considered significant.

## Results

### Effect of H_2_O_2_ on caveolin 1 expression level

**Fig 1:**
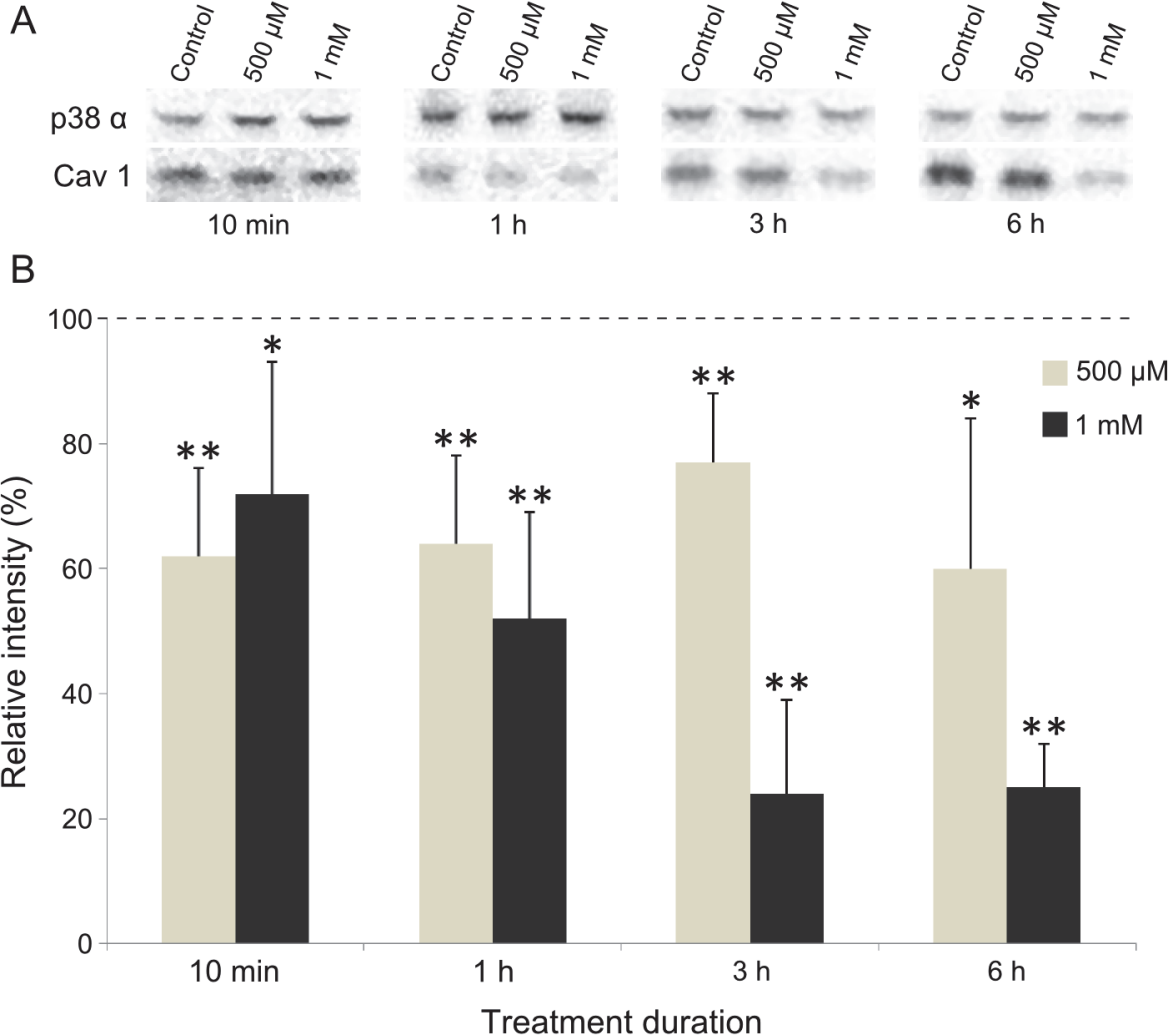
Effect of H_2_O_2_ on caveolin 1 expression level. Cells were treated with 500 or 1000 μM H_2_O_2_ for different time. Proteins were extracted and 15 μg was subjected to SDS-PAGE. Immunodetection was carried out using specific antibodies for caveolin 1 and p38α, which was used as a loading control (A). The level of caveolin 1 (cav1) was quantified and expressed as a percentage of the level obtained in the untreated control cells (—-). Bars on the graph represent the SEM (B). *Significantly different from the control (*P* < 0.05), **significantly different from the control (*P* < 0.01).

Caveolin 1 has been shown to interact with numerous oxidative enzymes [27, 28] and could therefore be involved in the regulation of oxidative stress-induced pathways. To answer that question, we first decided to determine if caveolin 1 expression level was somehow modified by H_2_O_2_ treatment. Western blots were carried out using protein extracts from C_2_C_12_ cells submitted (or not) to various concentrations of H_2_O_2_. As shown in Fig. 1, addition of 500 and 1000 μM H_2_O_2_ to the culture medium of the cells markedly decreased the expression level of caveolin 1. The protein amount was about 65% of the untreated sample as soon as 10 min after addition of 500 μM H_2_O_2_. This reduced level stayed roughly unchanged for longer time exposures. A more drastic decrease of caveolin 1 concentration was observed after 1000 μM H_2_O_2_ treatment, from 72 to 25% of the control between 10 min and 6 h, respectively (Fig. 1). Lower doses (100 and 250 μM) of hydrogen peroxide were tested under the same conditions and showed no significant effect on caveolin 1 level (data not shown). These results suggest that caveolin 1 is a target of oxidative damages in proliferating mouse myoblasts.

### Effect of H_2_O_2_ on cell viability

To determine the effect of H_2_O_2_ on cell viability, the PrestoBlue Cell Viability Assay was used as described in Materials and Methods. As shown in Table 1, 500 and 1000 μM H_2_O_2_ were tested and 1000 μM H_2_O_2_ had the most significant effect on cell death: 82% viability after 1 h, 56% after 3 h, reaching only 8% after a 6 h treatment. On the other side, after 500 μM H_2_O_2_ addition, 6 h incubation was necessary to observe a significant reduction of cell viability. These results indicate that the caveolin 1 decrease induced by 500 μM H_2_O_2_ was measured in viable cells (see Table 1 and Fig. 1), meaning that oxidative stress does not need to strongly affect myoblasts to trigger measurable effects on caveolin 1 protein level.

**Table 1:** Effect of H_2_O_2_ on cell viability.

H_2_O_2_ (μM)	500				1000			
Time	10 min	1 h	3 h	6 h	10 min	1 h	3 h	6 h
**Cell viability**(% total)	101	99.4	90.8	77.2*	93.2	81.7*	56*	8.1*
*SEM*	*2.25*	*10.7*	*11.1*	*12.7*	*4.75*	*6.75*	*8*	*0.57*

*Significantly different from the untreated control sample (*P* < 0.05).

### Oxidative stress induction after H_2_O_2_ treatment

Although H_2_O_2_ has already been used to induce oxidative stress in skeletal muscle cells [24, 29], we assessed that our experimental conditions promoted significant intracellular oxidation. Reactive oxygen species (ROS) were measured with the cell-permeant fluorogenic dye 2’, 7’-dichlorofluorescein diacetate (DCFDA). After cells were incubated with 500 or 1000 μM H_2_O_2_, intracellular ROS content increased 4 or 6 times, respectively, as compared with the untreated controls (Table 2). The reactive oxygen species accumulated as soon as 10 min after H_2_O_2_ addition. After 1 h incubation with H_2_O_2_, an increase of ROS intracellular level was observed, without being statistically different from 10 min (data not shown).

**Table 2:** Effect of H_2_O_2_ on ROS and carbonyl contents.

H_2_O_2_ (μM)	0	500	1000
**ROS content** (Fluorescence intensity arbitrary unit)	38884	171995*	233764*
*SEM*	*5998*	*44711*	*69976*
**Carbonyl content** (nmol/mg)	0.98	3.74*	4.36*
*SEM*	*0.68*	*0.85*	*0.53*

*Significantly different from the untreated control sample (*P* < 0.05).

In the meantime one of the multiple consequences of intracellular ROS accumulation was evaluated by the quantification of the carbonyl content, an indicator of protein oxidation [30]. As shown in Table 2 the amount of carbonyl groups was 0.98 nmol/mg of protein in control cells, and reached 3.74 and 4.36 nmol/mg of protein after 500 or 1000 μM H_2_O_2_ addition, respectively.

Both of these experiments indicated that a non-toxic H_2_O_2_ treatment (500 μM) of myoblasts was sufficient to induce a significant intracellular oxidative stress.

### H_2_O_2_-induced degradation of caveolin 1 via the proteasome pathway

**Fig 2:**
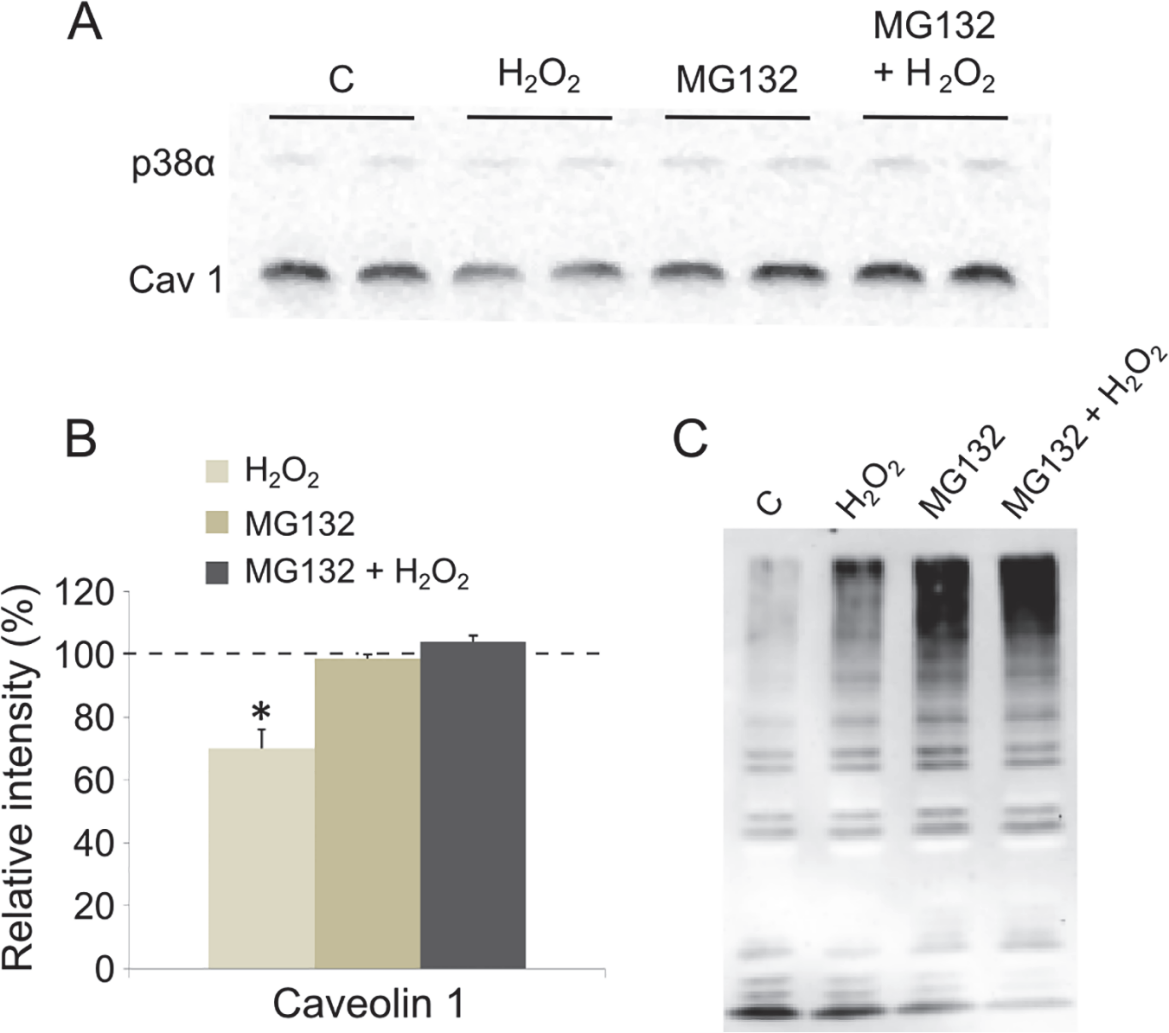
Effect of MG132 on H_2_O_2_-induced caveolin 1 decrease. Cells were pre-incubated for 3 h with MG132 (50 μM) before addition of 500 μM H_2_O_2_. After 10 minutes, cells were collected and protein extracts (15 μg) were loaded onto SDS-PAGE. Immunodetection was carried out using specific antibodies for caveolin 1 (cav1) and p38α, which was used as a loading control (A). The level of caveolin 1 was quantified and expressed as a percentage of the level obtained in the untreated control cells (—-) (B). Immunodetection was also carried out to monitor polyubiquitinylated protein accumulation using anti-ubiquitin antibodies (C). C: control. Bars on the graph represent the SEM. *Significantly different from the control (*P* < 0.05).

Caveolin 1 expression level decreased very promptly (10 min) after H_2_O_2_ addition to the cells (Fig. 1). This quick disappearance rather favors a degradation of the protein than a transcriptional down-regulation. The proteasome degradation pathway has been shown to be responsible for damaged proteins rapid elimination including oxidized proteins [31]. Therefore, we used a specific proteasome inhibitor (MG132) and measured its effect on caveolin 1 level with and without H_2_O_2_ treatment. Prior to this, we checked that MG132 was not toxic for the cells (Table 3). As shown in Fig. 2, when cells were incubated with 50 μM MG132 3 h before adding 500 μM H_2_O_2_, caveolin 1 level was not different from the untreated control cells. Interestingly, we were able to show polyubiquitinylated protein accumulation in cells incubated with MG132, confirming the inhibition of proteasome activity (Fig. 2C). Thus, pre-incubation with a specific proteasome inhibitor prevented H_2_O_2_-induced caveolin 1 degradation. In addition treatment with MG132 alone did not affect the protein amount. These data indicate that caveolin 1 is likely rapidly degraded by the proteasome-dependent pathway after H_2_O_2_ treatment of the cells.

**Table 3:** Effect of MG132 on cell viability.

H_2_O_2_	−	−	+
MG132	−	+	+
DMSO	+	−	−
**Cell viability**(% total)	96.6	97.3	103.5
*SEM*	*3*.*5*	*2*.*3*	*3*

### Caveolae assembly and membrane relative distribution were unaffected by H_2_O_2_

**Fig 3:**
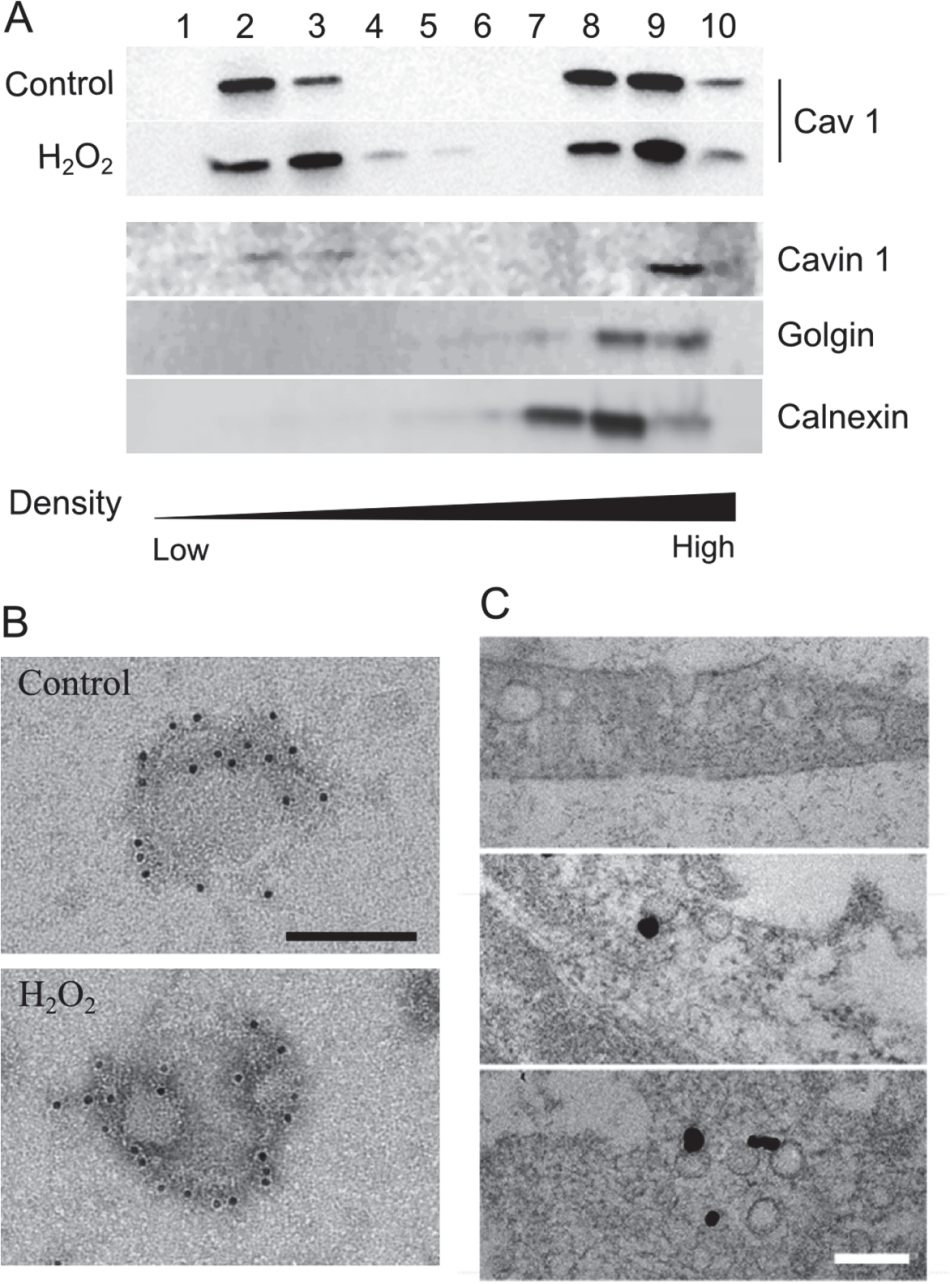
Effect of H_2_O_2_ on caveolae isolated through flotation gradient and identification of caveolae in C_2_C_12_ myoblasts. Myoblasts were incubated one hour with 500 μM H_2_O_2_. Cell extracts were fractionated by flotation trough a discontinuous sucrose gradient as described in materials and methods. Western blotting of fractions 1 to 10 was carried out with anti-caveolin 1 antibodies. Cavin 1, Golgin and Calnexin were immunoblotted to identify caveolae, golgi and RE specific fractions (A). Fraction 2 isolated from the flotation gradient was processed for immune electron microscopy using anti-caveolin 1 primary antibodies and 10 nm gold-conjugated protein A (B). Myoblasts were processed for (immune) electron microscopy as described in materials and methods. Representative electron micrographs show the morphology of caveolae (top panel) and caveolin 1 enriched “open” vesicular structures at the plasma membrane (middle) or “closed” in the cytosol (bottom panel) (C). Scale bar = 100 nm.

We aimed to morphologically identify caveolae in proliferating myoblasts. Although caveolin 1 is expressed in undifferentiated muscle cells, its oligomerization into caveolae still remains unclear [32, 33]. In a first set of experiments, we isolated caveolae and characterized them by transmission electron microscopy (TEM) (Fig. 3). The distribution of caveolin 1 was determined by western-blotting within low- and high-density membrane fractions obtained by sucrose density fractionation. As shown in Fig. 3A, caveolin 1 was localized in the low density fractions (2 and 3) corresponding to detergent-resistant membranes (DRMs) known to contain caveolae as confirmed by the co-localization with cavin 1 [34, 25]. An aliquot of fraction 2 was observed by TEM as described in Materials and Methods. Round-shaped vesicles with an average size of 80 / 100 nm and clearly delimited by lipid membranes were observed in this low-density fraction of the gradient. Among these, some clearly exhibited a dense immunogold labelling for caveolin 1 (Fig. 3B). Caveolin 1 was also present in fraction 8 and 9 (Golgi and Endoplasmic Reticulum), and in the cell pellet (lane 10, Fig. 3A). In a second set of experiments, myoblasts were directly observed by TEM. As shown in Fig. 3C, membrane invaginations and intracellular vesicles in the range of ~ 60 nm were clearly visible. Some of them were decorated with gold-labelled anti-caveolin 1 antibodies. Altogether these experiments indicate that caveolin 1 can assemble into caveolae in C_2_C_12_ myoblasts.

**Fig 4:**
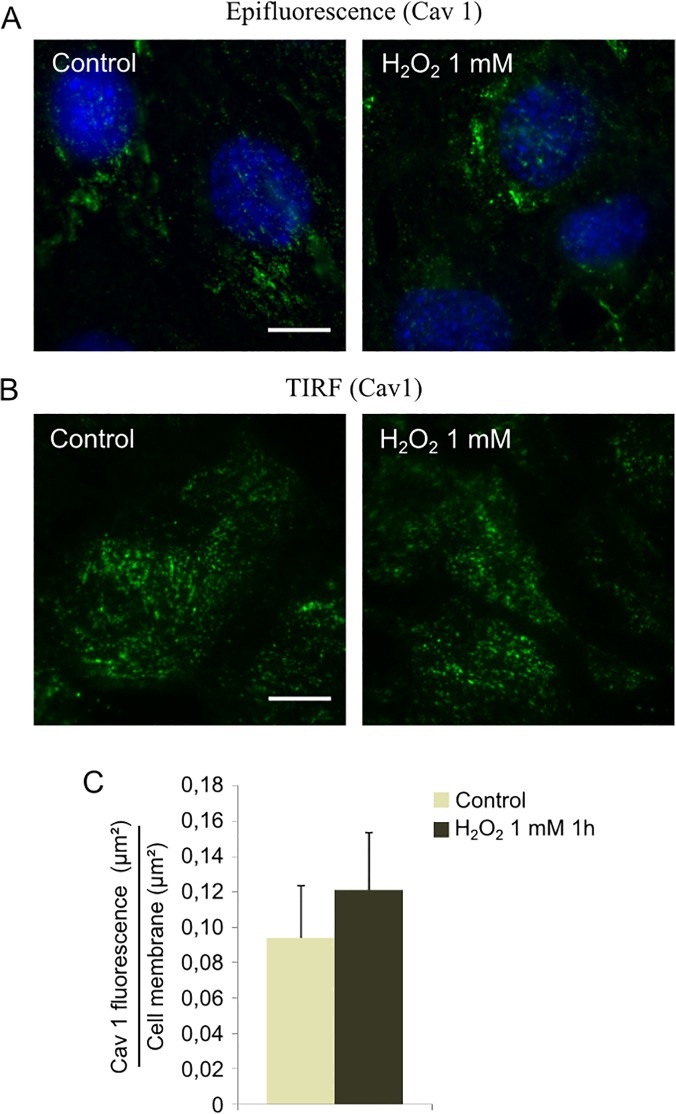
Effect of H_2_O_2_ on caveolin 1 localization. Cells were treated with 1 mM H_2_O_2_ during 1h. Caveolin 1 localization in C_2_C_12_ cells was determined by immunofluorescence labelling (A) or TIRF imaging (B). The ratio between membrane area with caveolin 1 labeling and total membrane area was expressed after TIRF images analysis (C). Bars on the graph represent the SEM. Scale bar = 10 μm.

As previously demonstrated caveolin 1 is affected by oxidative stress induction (Fig. 1). Since the protein is required for caveolae assembly, we wondered whether a decrease in caveolin 1 could impair the formation of caveolar vesicles after H_2_O_2_ treatment. Myoblasts were incubated with 500 μM H_2_O_2_ for one hour, and caveolin 1 was quantified by western blot in the different fractions isolated by sucrose density gradient. As shown in Fig. 3A, the low density caveolae-enriched fractions (2 and 3) contained similar caveolin 1-specific signal with or without H_2_O_2_. Aliquots of fraction 2 (control and H_2_O_2_-treated) were characterized by TEM and no obvious difference of labelling could be detected either. In parallel, epifluorescence microscopy experiments were carried out as described in Material and Methods. Caveolin 1 specific fluorescence did not significantly change after H_2_O_2_ addition to the cells (Fig. 4A). To specifically address the effect of H_2_O_2_ on membrane caveolae, we quantified the relative abundance of caveolin 1 by TIRF microscopy. As shown in Fig. 4B and C, oxidative stress had no significant effect on caveolin 1 distribution at the plasma membrane of myoblasts.

According to these data, although H_2_O_2_ treatment decreased caveolin 1 level, it had no significant effect on caveolae assembly and membrane relative distribution in C2C12 myoblasts.

### Influence of H_2_O_2_ treatment on caveolae functions

We next asked whether H_2_O_2_ could have consequences on two classical caveolae-mediated functions: endocytosis and mechanosensing [12, 35].

**Fig 5:**
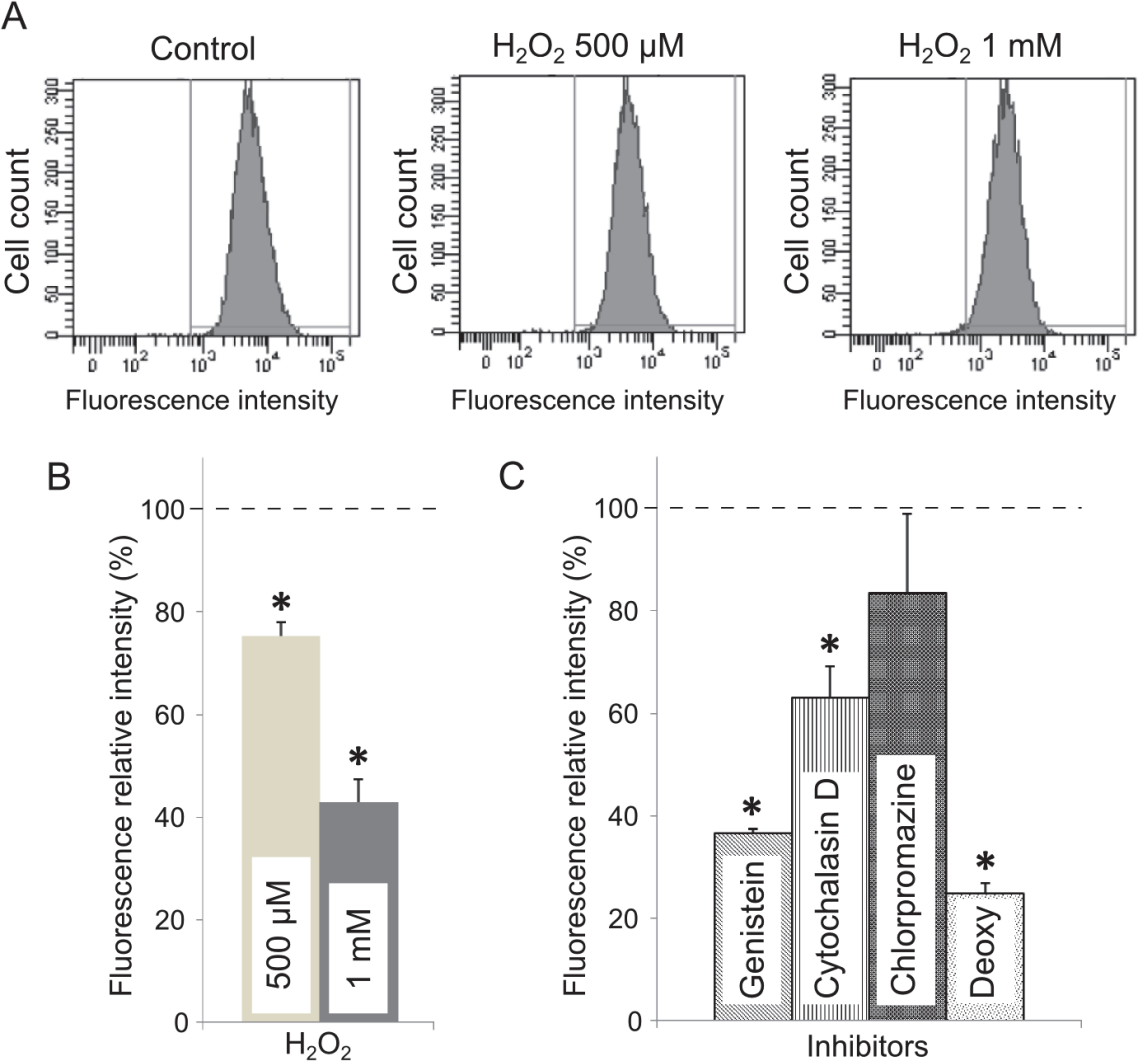
Effect of H_2_O_2_ on caveolae-dependent endocytosis. Cells were incubated with 0.25 μM of Bodipy-lactosylceramide after treatments with H_2_O_2_ or specific inhibitors of endocytic pathways as described in materials and methods. Cells were analyzed by flow cytometry (λ_exc_ = 488 nm) including at least 10 000 cells by condition (A). The mean cellular fluorescence intensity was expressed as a percentage of the fluorescence measured in the untreated control cells (—-) (B and C). Bars on the graph represent the SEM. *Significantly different from the control sample (*P* < 0.01).

To study the effect of H_2_O_2_ treatment on caveolae-specific endocytosis in C2C12 myoblasts, the cellular uptake of fluorescent Bodipy Lactosylceramide was quantified by flow cytometry analysis. Lactosylceramides have been shown to be specifically internalized in cells by caveolae [36, 37]. As shown in Fig. 5C, we confirmed that genistein, which inhibits caveolae-dependent endocytosis [38], significantly reduced Bodipy LacCer internalization. Chlorpromazine, an inhibitor of clathrin-dependent endocytosis, had no effect on intracellular fluorescence intensity. Cytochalasin D however, known to impair actin dynamics and thereby caveolae endocytosis, partially inhibited lactosylceramide uptake (Fig. 5C, 63% of the control). As expected, when all energy-dependent mechanisms were switched-down by ATP depletion (see “Deoxy” condition Fig. 5C), lactosylceramide uptake was very low as compared to untreated cells. After H_2_O_2_ addition, the intracellular relative fluorescence intensity was significantly lowered (75 and 43% of the controls after addition of 500 and 1000 μM H_2_O_2_, respectively, Fig. 5). These data indicate a net decrease of caveolae-dependent endocytosis of the fluorescent sphingolipid.

**Fig 6:**
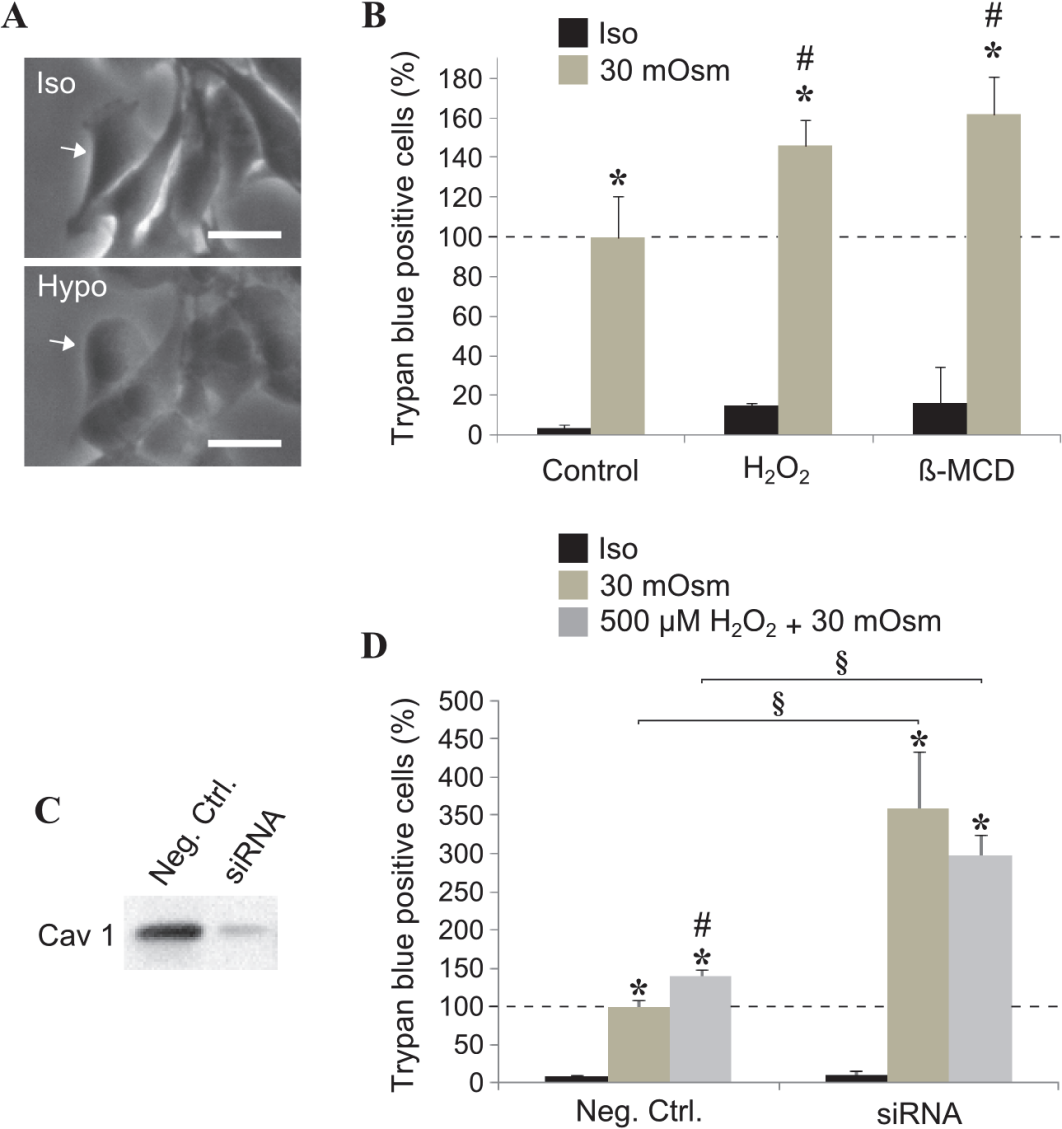
Effect of H_2_O_2_ on cell membrane rupture in hypo-osmotic conditions. Myoblasts were grown in iso- (300 mOsm) or hypo-osmotic medium (30 mOsm). Cell swelling was observed by light microscopy after 5 min (A). Cells were pre-incubated (or not) with H_2_O_2_ or β-methyl-cyclodextrin (β-MCD), left in iso- or hypo-osmotic medium for 30 min and stained with Trypan Blue (B). The cells were treated with a caveolin 1 siRNA or a negative control as described in material and methods. The reduction of caveolin 1 expression was confirmed by western blot (C) and the above swelling protocol was applied to the cells pre-incubated (or not) with 500 μM H_2_O_2_ (D). A minimum of 100 cells were counted by experiment and the Trypan blue positive cells were expressed as a percentage of the cells submitted to hypo-osmotic stress (—- in B and D). Bars on the graph represent the SEM. *Significantly different from the relative iso-osmotic sample (*P* < 0.05). ^♯^ Significantly different from the untreated related control (without H_2_O_2_, *P* < 0.05). ^§^ Significantly different from the negative control (*P* < 0.05). Scale bar = 30 μm.

According to Sinha and co-workers, caveolae would be responsible for membrane tension buffering under hypo-osmotic conditions [15]. We thus submitted myoblasts to hypo-osmotic shock (30 mOsm) and measured membrane rupture 30 min later by Trypan blue staining. As soon as 5 min after changing the osmolarity in the culture medium, cell swelling could be easily observed by light microscopy (compare Iso and Hypo, Fig. 6A). The resulting increase in membrane tension led to the rupture of myoblast membranes in 30 minutes (Fig. 6B). Under milder condition (150 mOsm), cell swelling could be followed under the light microscope without causing any significant damage on myoblast membranes (data not shown). When oxidative stress was applied to the cells 10 min beforehand, membrane rupture was significantly increased after 30 min in 30 mOsm medium (Fig. 6B). As a positive control cells were treated with β-methylcyclodextrin (β-MCD), a drug widely used to deplete cholesterol and disrupt caveolae structures [39]. As shown in Fig. 6, β-MCD treatment significantly impaired cell resistance to osmotic shock in a similar way than H_2_O_2_ treatment. Although β-MCD can impair caveolae formation, the drug might have a more pleiotropic effect in the cells. Therefore we used a specific siRNA and significantly reduced caveolin 1 expression (80% inhibition, Fig. 6C). As shown in Fig. 6D, in these conditions, the cells were less resistant to membrane tension increase.

Both of the abovementioned experiments indicated that oxidative stress induction impairs two caveolae-dependent cell processes: endocytosis and membrane resistance to cell swelling.

## Discussion

In this paper, we show for the first time that caveolin 1 is degraded by the proteasome after oxidative stress induction in mouse myoblasts. Oxidative damage has been proposed as one of the major contributors to skeletal muscle aging. When localized in satellite cells, it could be responsible for the failure of myogenic regenerative process observed during muscle ageing [40]. Caveolin 1 has been linked to oxidative-regulating pathways. The protein interaction with several oxidative enzymes (eNOS, NOX…) is known to inhibit their specific activities [41]. More recently, several authors suggested that caveolin 1 could be responsible for negatively regulating antioxidant defenses in fibroblasts through its interaction with Nrf2 transcription factor [20, 42]. However, the effect of oxidative stress on the protein expression level itself still remained controversial and clearly dependent on the cell type. While Hsieh et *al*. showed that H_2_O_2_ treatment decreased caveolin 1 expression in cardiomyocytes, Dasari et *al*. demonstrated the opposite effect of comparable doses of oxidant in epithelial cells [43, 44]. Here, we added 500 μM or 1000 μM H_2_O_2_ directly to the culture medium of myoblasts as currently done [29, 45]. H_2_O_2_ is known to freely diffuse across cellular membranes and give rise to intracellular reactive oxygen species especially in skeletal muscle [46]. Nonetheless we controlled the induction of oxidative stress inside our cells by measuring ROS accumulation and their consequences on proteins. ROS significantly accumulated inside the cells as soon as 10 min after H_2_O_2_ addition to the culture medium. Moreover, protein carbonyls, one of the most current cellular consequences of oxidative stress [30], increased 3 to 4 times after H_2_O_2_ addition (Table 2). In a previous study carried out in rat skeletal muscle, we described similar increases in carbonyl content during aging [47]. Our conditions of treatment thus represent a good cellular model for studying the mechanisms involved in skeletal muscle aging. Interestingly, we were able to induce a significant oxidative stress in proliferating myoblasts without affecting the viability of the cells (see Table 1, 500 μM 10 min to 3h, and 1000 μM 10 min).

Most importantly, caveolin 1 expression level was very rapidly affected after H_2_O_2_ addition to the culture medium (10 min, Fig. 1). The protein level decreased by almost 30%, similarly to what Hsieh et *al*. recently described in cardiomyocytes [43]. These results suggest that the protein is directly, but most remarkably, very quickly targeted by oxidative stress in proliferating mouse myoblasts. According to several authors, such a rapid elimination of a protein could be imputable to the proteasome, especially after oxidative injuries [31, 48]. Here we showed that H_2_O_2_ had no longer effect on caveolin 1 expression level when cells were pre-incubated with a specific proteasome inhibitor (MG132) (Fig. 2). These data confirm that caveolin 1 is rapidly degraded by the proteasome-dependent pathway after H_2_O_2_ treatment of the cells. As aforementioned, caveolin 1 would behave as a negative regulator of cellular antioxidant defenses. In the light of the present data, the early degradation of the protein after stress induction could then trigger the cellular antioxidant defenses.

During skeletal muscle differentiation and/or regeneration, caveolin 1 and 3 are sequentially expressed [49]. Caveolin 1 is highly expressed in proliferating myoblasts and *cav-1* gene becomes switched-off as the cells begin to fuse into multinucleated myotubes. In parallel, caveolin 3 expression is up-regulated as differentiation of the myogenic cells occurs. Both proteins are however absolutely necessary for caveolae biogenesis and structuration [50, 51]. Mutations in the *cav-3* gene or alterations of caveolin 3 expression level have been closely associated with major skeletal muscle dysfunctions and pathologies (LGMD1C, DMD, …) [17, 18]. On the opposite, the consequences of caveolin 1 deregulation has never been clearly studied in muscle, although the protein has been largely linked to several major diseases in other tissues: breast cancer, atherosclerosis…[52, 53]. Here we observed a significant loss of caveolin 1 in proliferating myoblasts after intracellular stress induction. We next asked whether the decrease in caveolin 1 would lead to a loss of caveolae. Surprisingly, we were not able to evidence any effect of H_2_O_2_ treatment on caveolae membrane density by TEM (Fig. 3), as shown before in cardiac myocytes for instance [54]. Caveolae were however too sparse at the membrane in our cell type compared to cardiac tissue. We therefore combined biochemical and microscopic approaches to access the information.

First, we looked at the ability of caveolae to assemble normally in cells submitted to oxidative stress. Caveolin-containg fractions were isolated by sucrose density fractionation from myoblasts treated with H_2_O_2_. As shown in Fig. 3A, caveolin 1 was enriched in low-density fractions (2 and 3) of the gradient, as confirmed by the co-localization of caveolin 1 and cavin 1. Remarkably, H_2_O_2_ addition to the cells did not significantly affect the fractionation of caveolin 1 between low- and high-density fractions. Whereas these data cannot rule out that the caveolae relative amount was unaffected by H_2_O_2_, they suggest however that caveolae were still able to assemble normally regardless of H_2_O_2_ addition. Cellular material obtained in fraction 2 was also observed by TEM after caveolin 1 specific labelling. As clearly seen on the pictures, H_2_O_2_ did not affect the size of the vesicles, neither changed the relative abundance of caveolin 1 per caveolae (Fig. 3B).

Second, TIRF microscopy was carried out to follow the effect of oxidative stress on caveolin 1 relative abundance at the plasma membrane. The specific fluorescence of the protein detected at the membrane of the myoblasts stayed unchanged whether the cells were treated with 1 mM H_2_O_2_ or not (Fig. 4). The discrepancy between the strong signal monitored by TIRF microscopy and the few caveolae observed in C_2_C_12_ myoblasts by TEM could be explained by the conformation of the caveolae itself. Although caveolae can be present at the plasma membrane, they might require an activation step to harbor the omega shape classically detected by TEM [55].

Altogether these data indicate that H_2_O_2_-induced caveolin 1 degradation had no significant consequence on caveolae assembly and localization at the plasma membrane.

We next focused on the consequences of H_2_O_2_ treatment on caveolae-mediated functions. We studied two caveolae-dependent cell functions: endocytosis and mechanosensing [12, 35]. Lactosyl-ceramides have been shown to be internalized by caveolae [36, 37]. We therefore monitored caveolae endocytosis with this specific probe in an assay where internalization of the fluorescent Bodipy coupled lactosyl-ceramide was measured by flow cytometry analysis after H_2_O_2_ treatment. The data nicely showed a net decrease of the intracellular fluorescence as H_2_O_2_ increased (Fig. 5). As shown in Fig. 5C, when caveolae-specific endocytosis was inhibited (genistein), the fluorescence intensity significantly decreased in the cells, confirming the specificity of the assay. We also blocked actin dynamics with cytochalasin D, and we observed a partial inhibition of the ceramide uptake (Fig. 5C) in agreement with published data on cytochalasin D impairing caveolae internalization through actin depolymerization [56]. According to our data, oxidative stress induction is likely to negatively impact caveolar endocytosis.

Another function of caveolae has recently emerged in the literature: the maintenance of plasma membrane through the constitution of a caveolae “membrane reservoir” buffering membrane tension variations [15, 54]. In our experiments, we studied the effect of oxidative stress on membrane tension buffering under hypo-osmotic conditions. Proliferating myoblasts were submitted to hypo-osmotic shock and membrane rupture was measured by the penetration of Trypan blue dye. As shown in Fig. 6, reducing the osmolarity in the culture medium from 300 to 30 mOsm caused the rupture of myoblast membranes in 30 min. In another experiment, we incubated the cells in a 150 mOsm medium and had no effect on plasma membranes, although the cell swelling could be microscopically observed (data not shown). As mentioned above, caveolae are able to supply the cells with “new” membranes when necessary, we could therefore imagine that 30 mOsm would overcome the ability of caveolae to maintain cell membrane integrity. When H_2_O_2_ was applied to the cells 10 min beforehand, membrane rupture was significantly increased after 30 min in 30 mOsm medium suggesting that caveolae integrity and/or number were affected and could not guarantee mechanical resistance anymore (Fig. 6B). Similar result was obtained with methyl-beta-cyclodextrin, a cholesterol-complexing drug currently employed to disrupt caveolae inside the cells [39, 54], corroborating the role of caveolae in buffering membrane tension. To further confirm the effect observed with methyl-beta-cyclodextrin, we directly inhibited caveolin 1 expression with siRNA to have a more specific control. As shown in Fig. 6C, we reduced caveolin 1 expression by 80%. In this condition, myoblasts were three times more sensitive to the hypo-osmotic shock (Fig. 6D). Remarkably, when H_2_O_2_ was added during the osmotic stress, the amount of Trypan blue positive cells stayed unchanged indicating that osmotic swelling-induced membrane rupture was only imputable to a breach in caveolae. Therefore, we unequivocally showed that oxidative stress specifically impaired caveolae contribution to plasma membrane tension buffering (Fig. 6D).

In conclusion, our results showed that caveolin 1 is degraded by the proteasome-dependent pathway soon after oxidative stress induction in myoblasts. However, and despite the decrease in caveolin 1 level, the assembly and distribution of caveolae at the plasma membrane were not significantly affected by oxidative stress. Endocytosis and membrane resistance to hypo-osmotic challenge, two caveolae-specific cellular functions, were significantly impaired. Taken together, these data would suggest that the caveolae-dependent pathway could take part to the regulation of oxidative stress in skeletal muscle. Caveolin 1 level could be involved for instance in the activation of the cellular defenses or regulate regenerative process, several important mechanisms impaired during muscle aging. We also clearly showed that H_2_O_2_ treatment deeply affected caveolae functions (endocytosis and membrane tension buffering). Further experiments need to be carried out to understand the exact mechanisms linking the caveolae-dependent pathway and oxidative stress during muscle aging.
